# Activation of the kynurenine pathway identified in individuals with covert hepatic encephalopathy

**DOI:** 10.1097/HC9.0000000000000559

**Published:** 2024-11-15

**Authors:** Georgia Zeng, Shivani Krishnamurthy, Ananda Staats Pires, Anna Guller, Joga Chaganti, Nway Tun, Ian Lockart, Sara Montagnese, Bruce Brew, Gilles J Guillemin, Mark Danta, Benjamin Heng

**Affiliations:** 1 Department of Gastroenterology and Hepatology, St Vincent’s Hospital, Sydney, Australia; 2 School of Clinical Medicine, St Vincent’s Healthcare Campus, Faculty of Medicine, UNSW Sydney, Sydney, Australia; 3 Macquarie Medicine School, Faculty of Medicine, Health and Human Sciences, Macquarie University, Sydney, Australia; 4 Computational Neurosurgery (CNS) Laboratory, Macquarie Medicine School, Faculty of Medicine, Health and Human Sciences, Macquarie University, Sydney, Australia; 5 Department of Medical Imaging, St Vincent’s Hospital, Sydney, Australia; 6 Department of Medicine, Padova University Hospital, Padova, Italy; 7 Department of Chronobiology, Institute for Sustainability, Faculty of Health and Medical Sciences, University of Surrey, Guildford, UK; 8 Department of Neurology, St Vincent’s Hospital, Sydney, Australia; 9 Institut Pertanian Bogor (IPB) University, Bogor, Indonesia

**Keywords:** cirrhosis, hepatic encephalopathy, kynurenine pathway, neurotoxicity

## Abstract

**Background:**

HE is a neuropsychiatric complication of liver disease characterized by systemic elevation in ammonia and proinflammatory cytokines. These neurotoxins cross the blood-brain barrier and cause neuroinflammation, which can activate the kynurenine pathway (KP). This results in dysregulated production of neuroactive KP metabolites, such as quinolinic acid, which is known to cause astrocyte and neuronal death. Our aim was to compare KP activity between patients with covert HE (CHE), patients without encephalopathic cirrhosis (NHE), and healthy controls (HCs).

**Methods:**

This was a single-center prospective cohort study conducted between 2018 and 2021 at St Vincent’s Hospital, Sydney. Overall, 13 patients with CHE, 10 patients with NHE, and 12 with HC were recruited. Patients with cirrhosis were diagnosed with CHE if they scored ≤−4 on the Psychometric Hepatic Encephalopathy Score. KP metabolite levels were quantified on plasma samples via HPLC and gas chromatography/mass spectrometry. One-way Kruskal-Wallis test was used to compare the expression levels of KP enzymes.

**Results:**

KP was highly activated in patients with cirrhosis, demonstrated by higher levels of activity in the rate-limiting enzymes, indoleamine 2,3-dioxygenase, and tryptophan-2,3-dioxygenase in both CHE (65.04±20.72, *p*=0.003) and patients with NHE (64.85±22.10, *p*=0.015) compared to HC (40.95±7.301). Higher quinolinic acid concentrations were demonstrated in CHE (3726 nM±3385, *p*<0.001) and patients with NHE (1788 nM±632.3, *p*=0.032) compared to HC (624 nM±457). KP activation was positively correlated with inflammatory marker C-reactive protein in patients with CHE (R_s_=0.721, *p*≤0.01).

**Conclusions:**

KP is highly activated in patients with CHE, resulting in heightened production of neurotoxic metabolites. Dysregulation of the pathway is demonstrable in patients who do not yet show clinical signs of neurocognitive impairment. Therapeutic agents that modulate KP activity may be able to alleviate symptoms of patients with CHE.

## INTRODUCTION

HE is a critical complication of liver disease and/or portosystemic shunting that is often underdiagnosed in clinical settings. Overt hepatic encephalopathy (OHE) is defined by the presence of at least asterixis or temporal disorientation, corresponding to grades II–IV by West Haven criteria. OHE episodes, reported in 30%–50% of patients with cirrhosis, are associated with negative impacts on quality of life and survival.^1^ Milder forms of neurocognitive impairment in patients with cirrhosis are defined as covert hepatic encephalopathy (CHE), which has also been associated with higher risk of hospitalizations and OHE episodes.^2^

**FIGURE 1:**
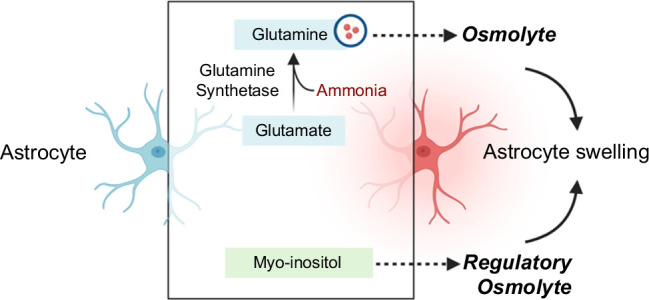
A simplified schematic depicting the relationship between select brain metabolites and cerebral edema in HE. In the brain, ammonia is disposed of through the synthesis of glutamine from glutamate via the enzyme glutamine synthetase in astrocytes. In HE, there is an increase in glutamine and depletion of myoinositol, respectively. The increase in glutamine results in astrocyte swelling due to its osmolytic properties, while the efflux of myoinositol from astrocytes is a compensatory mechanism.

Although the exact pathological mechanisms contributing to the development of HE are multifaceted, elevated systemic concentrations of neurotoxins such as ammonia and inflammatory cytokines have been identified as key characteristics of this disease. Acute or chronic liver dysfunction compromises the capacity for ammonia removal via the urea cycle, leading to systemic hyperammonemia.^3^ Other crucial sites for ammonia metabolism include the brain, kidneys, and skeletal muscle.^4^,^5^ However, the brain lacks a complete urea cycle; thus, excess ammonia must be disposed of during the conversion of glutamate to glutamine via the glutamine synthetase pathway, a process localized in astrocytes. The production of glutamine correlates with the increased accumulation of ammonia in the brain, leading to morphological change in astrocytes such as swelling once past the point of compensatory efflux of myoinositol.^6^ A simplified schematic depicting the relationship between these metabolites, as measured via brain magnetic resonance spectroscopy, and the cerebral edema of HE is demonstrated in Figure 1. The neuropsychiatric effects of hyperammonemia can be compounded by the systemic inflammatory response in HE,^7^ which is evidenced by higher levels of serum cytokines such as IL-6 and IL-18 in patients with encephalopathy compared to patients without encephalopathic cirrhosis.^8^ Higher levels of ammonia in the central nervous system are facilitated by an increased permeability of the blood-brain barrier (BBB) in this disease state, which has been observed in both animal bile duct ligation (BDL) models of HE^9^ and our study in patients with CHE.^10^ These findings may play an integral role in the capacity of systemic toxins to create a central nervous system environment of neuroinflammation in HE.^11^

**FIGURE 2:**
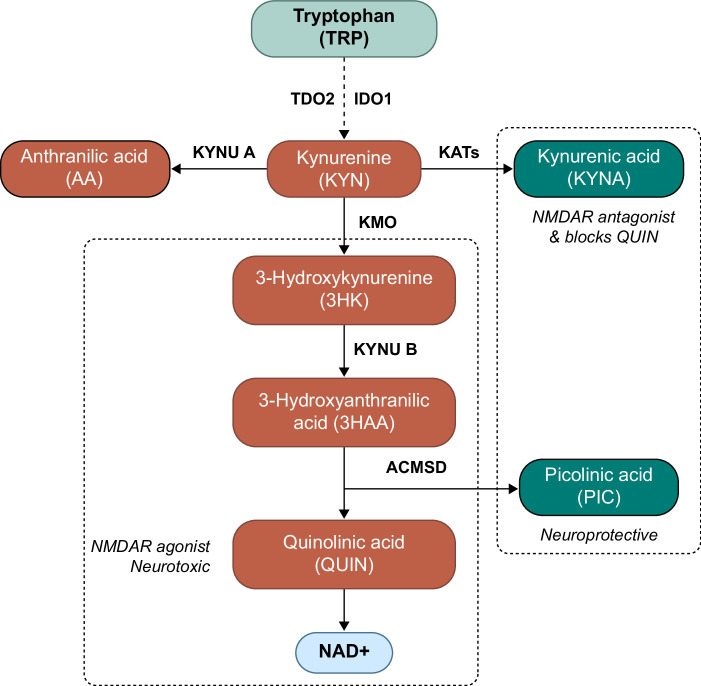
A schematic diagram of the KYN pathway. TRP ( highlighted in light green) is largely metabolized through the KP to produce NAD^+^ (highlighted in blue), an essential cellular energy factor. Tryptophan is metabolized to produce KYN, which then leads to the production of other metabolites, notably 3HK, KYNA, and AA. Highlighted in red is the neurotoxic branch of the kynurenine pathway, showing the conversion of KYN to 3HK and finally to QUIN, the key neurotoxic metabolite. The metabolites with neuroprotective properties, namely AA, KYNA, and PIC, are highlighted in dark green. Abbreviations: 3HAA, 3-hydroxy anthranilic acid; 3HK, 3-hydroxykynurenine; AA, anthranilic acid; ACMSD, amino-carboxymuconate-semialdehyde decarboxylase; KMO, Kynurenine monooxygenase; KYN, kynurenine; KYNA, kynurenic acid; KYNU, kynureninase; NMDAR, N-methyl-D-aspartate receptor; PIC, picolinic acid; QUIN, quinolinic acid; TRP, tryptophan.

Systemic and neuroinflammation can also dysregulate biochemical pathways, such as the kynurenine pathway (KP),^12^,^13^ which is responsible for metabolizing 95% of the essential amino acid tryptophan (TRP) in humans as a means to produce nicotinamide adenine dinucleotide (NAD+). The rate-limiting enzyme of the pathway, indoleamine 2,3-dioxygenase (IDO-1), can be induced by proinflammatory cytokines such as IFN-γ or TNF-α in the setting of systemic inflammation.^12^,^13^ This reduces the contribution of the alternative TRP degradation pathway via 5-hydroxytryptamine and 5-hydroxyindoleacetic acid,^14^ and augments the activity of the KP pathway. Kynurenine (KYN) is the central intermediate of the KP, which is then catabolized to generate anthranilic acid (AA), kynurenic acid (KYNA), and 3-hydroxykynurenine (3HK). The breakdown of 3HK generates several other neuroactive metabolites, including quinolinic acid (QUIN) and picolinic acid (PIC). A simplified schematic diagram of the KP pathway is represented in Figure 2. Among all of these, QUIN, 3HK, and KYNA have demonstrated neuroactive properties. Chronic production of QUIN can lead to dysfunction and death of astrocytes^15^ and neurons.^16^,^17^ QUIN directly binds to the N-methyl-D-aspartate receptor, which induces excitotoxicity, leading to cell death.^18^ 3HK is another neurotoxin that generates free radicals and potentiates the neurotoxicity of QUIN.^19^ On the other hand, KYNA blocks glutamate-mediated and nicotinic excitatory postsynaptic potentials^20^ but cannot entirely counteract QUIN binding during the disease state.^21^ QUIN is likely the most important KP metabolite in terms of neurotoxicity, and elevated levels have been demonstrated in neurological/cognitive diseases such as depression,^22^ suicidal behavior,^22^,^23^ schizophrenia^24^ and recently, cognitive impairment in patients with COVID-19.^25^ Additionally, disrupted production of NAD+, the end product of the pathway, may contribute to neuropsychiatric abnormalities in HE, given its role in adaptive stress responses and depletion in a number of neurodegenerative diseases.^26^

Considering neurotoxic potential of its downstream metabolites, dysregulation of the KP may contribute to the neuropsychiatric disturbances of HE, which can range from inattention and altered sleep patterns to somnolence and coma. Basile et al^27^ first reported high cerebrospinal fluid (CSF) and plasma levels of QUIN in animal models of fulminant hepatic failure and varying stages of HE, finding that levels were most elevated in stage IV HE. Jiang et al^28^ later demonstrated elevated levels of KYN, 3HK, and reduced KYNA in various brain regions after BDL, which correlated with increased serum proinflammatory cytokines and neuropsychiatric symptoms. Moreover, Jiang and colleagues demonstrated that introducing an inhibitor of rate ido enzyme IDO-1 reversed behavioral changes in the BDL rats, thus highlighting the therapeutic potential of modulating KP activity for patients with HE. Similarly, in a mouse model study, KYN levels became elevated 14 days post BDL, in correlation with the onset of microglial morphological changes.^29^ In regard to clinical studies, Clària et al^30^ have demonstrated dysregulation of the KP in patients with decompensated cirrhosis and acute-on-chronic liver failure, where high baseline KP activity was able to predict clinical course and mortality. Elevation of TRP degradation products in the CSF has also been demonstrated in patients with OHE.^31^ To our knowledge, this is the first clinical study to evaluate changes in the KP in patients with CHE who demonstrate more subtle neuropsychiatric alterations. The aim of our study was to compare serum levels of the KP metabolites in patients with CHE against patients without encephalopathic cirrhosis and healthy controls (HCs), and delineate any relationships between the KP metabolites and clinical parameters of liver disease. We hypothesize that there may be a correlation between the neuropsychiatric abnormalities of CHE and a skewed KP metabolite profile, which may further be associated with poor prognostic markers of liver disease.

## METHODS

### Study cohorts

This was a single-center prospective cohort study conducted between 2018 and 2021 at St Vincent’s Hospital in Sydney, Australia. We recruited 2 cohorts of participants: individuals with cirrhosis and CHE (n=13) and individuals with cirrhosis with no hepatic encephalopathy (NHE; n=10). Subjects who were under 18, diagnosed with OHE, diagnosed with acute intercurrent primary neurological pathology, suffering from claustrophobia, or any other contraindications to MRI were excluded from the study. These cohorts were compared against a set of HCs (HC; n=12), drawn from an external study cohort who had consented to the use of their data in future studies.

In individuals with cirrhosis, CHE was diagnosed using gold standard neuropsychological testing, specifically a Psychometric Hepatic Encephalopathy Score (PHES) of <−4, which has been standardized as the norm in other countries.^32^ The pen-and-paper-based PHES comprises 5 short tests: the Digit Symbol Test, Number Connection Tests A and B, Serial Dotting Test, and Line Tracing Test.^33^ Participants also underwent the computerized test of continuous reaction time (CRT), which assesses cognitive ability by measuring the participant’s reaction to auditory stimuli with a handheld trigger button, where a CRT index of <1.9 was abnormal.^34^ Patients with cirrhosis were classified as CHE if their PHES score was below −4 and as NHE if their PHES score was ≥−4, regardless of their CRT index.^35^ These tests were conducted for each participant in a quiet room with ample lighting, by the same experienced examiner. Demographic and clinical data were collected from each participant. Blood was collected for basic biochemistry and ammonia levels and to determine the MELD score and Child-Pugh (CP) score. An extra 10 mL whole blood sample was collected and stored, which was used to quantify the levels of the KP metabolites. Patients with CHE and NHE also completed the self-administered Chronic Liver Disease Questionnaire and underwent an assessment of hand grip strength using JAMAR Hydraulic Hand Dynamometer (Patterson Medical, Warrenville, IL). Additionally, patients with CHE and NHE underwent a 30-minute electroencephalogram in the Neurophysiological department, performed in an alert state and observing the effects of hyperventilation and photic stimulation, which was classified as encephalopathic or nonencephalopathic based on qualitative changes in the oscillatory dynamics of the cortical networks.^36^ Lastly, all participants with CHE and NHE underwent dynamic contrast-enhanced-MRI and magnetic resonance spectroscopy, the results of which are discussed in another study.^10^

This study was approved by the St Vincent’s Human Research Ethics Committee and performed in accordance with the Helsinki II Declaration, with the HREC study number HREC/18/SVH/57. All patients gave written informed consent before participating.

### Sample preparation for the analytical chemistry

Approximately 200 µL of the plasma samples collected from healthy volunteers, patients with HE and cirrhosis included in this study, were deproteinized by adding an equal volume of 10% (w/v) trichloroacetic acid followed by centrifugation at 4°C for 10 minutes at 12,000 rpm. Then, the deproteinized supernatants were filtered using 0.22 μm syringe filters (Millex, Merck) and transferred into analyzer vials.

### Quantification of KP metabolites by HPLC and ultra-HPLC

Ultra-HPLC system (Agilent 1290 Infinity, Santa Clara, CA) coupled with a temperature-controlled autosampler, column compartment, diode array detector, and fluorescence detector were used for concurrent quantification of TRP and 4 KP metabolites including KYN, 3HK, AA, and 3-hydroxylanthranilic acid (3HAA) as described.^37^ Approximately 10 µL of the filtered deproteinized sample was injected into the analyzer, and the metabolites were separated using 0.1 mM sodium acetate (pH 4.6) as the mobile phase, with an isocratic flow rate of 0.75 mL/min in an Eclipse Plus C18 reverse-phase column (2.1×150, 1.8 μm particle size; Agilent Technologies Inc., Santa Clara, CA) at a stable temperate of 40^o^C for 12 minutes. 3HK and KYN were detected using a UV detector with wavelength intensity set at 365 nM. TRP, 3HAA, and AA were detected using a fluorescence detector set at Ex/Em wavelength intensity of 280/438 for TRP and 320/438 for 3HAA and AA. A series of mixed standards of all 5 metabolites were used to draw a 6-point calibration curve into interpolate the quantity of the sample readout. The chromatogram output was analyzed using the Agilent OpenLAB CDS ChemStation (Edition C.01.04).

KYNA concentration in plasma samples was determined by HPLC (Agilent 1260 Infinity, Agilent Technologies Inc., Santa Clara, CA) and an Agilent ZORBAX Rapid Resolution High Definition C18, reversed phase (4.6×100 mm, 3.5 μm, Agilent Technologies, CA, USA). Mobile phase consisted of 95% of 50 mM sodium acetate and 50 mM zinc acetate, pH 5.2, and 5% HPLC grade acetonitrile. The flow rate was set at 1.00 mL/min with an isocratic elution. The identification of KYNA was performed by a fluorescence detector (G1321B xenon flash lamp, Agilent, CA) with an emission wavelength of 388 nm and an excitation wavelength of 344 nm. The results were calculated by interpolation using a six-point calibration curve. The chromatogram output was analyzed using the Agilent OpenLAB CDS ChemStation (Edition C.01.04).

### Quantification of KP metabolites by gas chromatography-mass spectrometry

The downstream KP metabolites, including PIC and QUIN were quantified using an Agilent 7890 A gas chromatography coupled with Agilent 5975 C mass spectrometry detector and Agilent 7693 A autosampler (Agilent Technologies Inc., Santa Clara, CA) as described.^37^ Fifty microliter of the filtered deproteinized samples mixed with deuterated internal standards respective to the metabolites of interest were derivatized for quantification. Approximately 1 µL of the derivatized sample was injected Agilent 7890A gas chromatography coupled with Agilent 5975C mass selective detector (Agilent Technologies Inc., Santa Clara, CA) and PIC and QUIN metabolites present in the samples were separated by a DB-5MS column, 0.25 mM film thickness, 0.25 mm×30 m capillary column (Agilent Technologies Inc., Santa Clara, CA). Final concentrations of PIC and QUIN were measured using Agilent gas chromatography/MSD ChemStation software (Edition 02.02.1431) to analyze the chromatogram outputs. A series of deuterated and non-deuterated standards for PIC and QUIN were used for a six-point calibration curve to interpolate the quantity of the sample readout.

### Statistical analysis

Statistical analysis of the KP data were performed and illustrated using Excel and GraphPad Prism 9 (GraphPad Software, Boston, MA). One-way Kruskal-Wallis test was used for the KP data analysis. The activity of the KP enzymes were inferred by a ratio of the enzyme product divided by its respective substrate. Differences in the concentration and activity of the KP metabolites and enzymes, measured between different comparison groups were considered significant if *p*≤0.05.

Correlation analysis was performed using SPSS version 26 (IBM Corp. Armonk, NY). Spearman correlation coefficient tests were used for the analysis of correlations between KP metabolites/enzyme activity and clinical data. Correlation data were graphically presented as a heatmap in color, where the tones of red represent positive correlations and the tones of green represent negative correlation. Black-colored font shows the data with a *p*≤0.05, whereas purple-colored italic font indicates the data with a *p*≤0.01. Correlations of moderate and strong certainty were reported in the main body of text.

## RESULTS

### Demographics of patients with CHE, NHE, and HCs

The demographics and baseline characteristics of the patient cohorts and healthy volunteers are shown in Table 1. Overall, the study included 13 patients with CHE (11 men and 2 women, aged 59.3 ± 6.2 y), 10 patients with NHE (6 men and 4 women, aged 64.6 ± 6.9 y), and 12 healthy volunteers (6 men and 6 women, aged 48.3 ± 7.5 y). The CHE cohort had more advanced liver disease defined by higher MELD score (17.5 ± 7.2 vs. 10.6 ± 4.4), CP score (7.9 ± 2.7 vs. 5.7 ± 1.3), and decompensation events. The increased decompensation events included ascites (61.5% vs. 20%), jaundice (30.7% vs. 0%), and previous HE (53.8% vs. 10%). Lactulose therapy was used by 69.2% of the CHE cohort and 10% of the NHE cohort. None were on rifaximin.

**TABLE 1:** Demographics and baseline characteristics of patient groups and healthy controls

	Healthy control (n=12) N (%)	NHE (n=10) N (%)	CHE (n=13) N (%)
Age (mean±SD)	48.3 (7.5)	64.6 (6.9)	59.3 (6.2)
Sex			
Male	6 (50)	6 (60)	11 (84.6)
Ethnicity			
Caucasian	12 (100)	10 (100)	13 (100)
Etiology			
Viral hepatitis	—	2 (20)	4 (30.7)
Alcohol	—	4 (40)	7 (53.8)
NAFLD	—	1 (10)	0 (0)
Viral hepatitis + alcohol	—	2 (20)	2 (15.4)
Alcohol + NAFLD	—	1 (10)	0 (0)
History of liver decompensation			
Ascites	—	2 (20)	8 (61.5)
Jaundice	—	0 (0)	4 (30.7)
Variceal bleed	—	3 (30)	2 (15.4)
Previous HE	—	1 (10)	7 (53.8)
Already on HE medications	—	1 (10)	9 (69.2)
Diagnostic scores (mean±SD)			
MELD score	—	10.6±4.4	17.5±7.2
Child-Turcotte-Pugh score	—	5.7±1.3	7.9±2.7
Chronic liver disease questionnaire score	—	162.5±30.4	118.5±40.3
Psychometric HE score	—	−2.1±2.1	−8.8±2.6
Continuous reaction time test index	—	2.13±0.62	1.58±0.54
Blood-based clinical data (mean±SD)			
Ammonia (μmol/L)	—	46.8±33.6	87.41±59.4
Sodium (mmol/L)	—	141.1±2.0	136.7±5.1
Creatinine (μmol/L)	—	90.8±33.1	89.9±24.6
Albumin (g/L)	—	38.2±5.2	33±8.8
Bilirubin (μmol/L)	—	22.2±15.2	61.7±63.5
Platelets (10^9^/L)	—	141.9±57.5	87.1±47.6
C-reactive protein	—	4.3±5.6	8.1±9.8
Erythrocyte sedimentation rate (ESR)	—	29.4±18.6	29.6±25.1
ALT (U/L)	—	31.4±10.8	34.2±21.8
AST (U/L)	—	46.8±21.6	65.8±38.7

Abbreviations: CHE, covert hepatic encephalopathy; ESR, erythrocyte sedimentation rate; NHE, no hepatic encephalopathy.

### IDO-1/tryptophan 2,3-dioxygenase enzyme activity is elevated in patients with CHE and NHE

**FIGURE 3:**
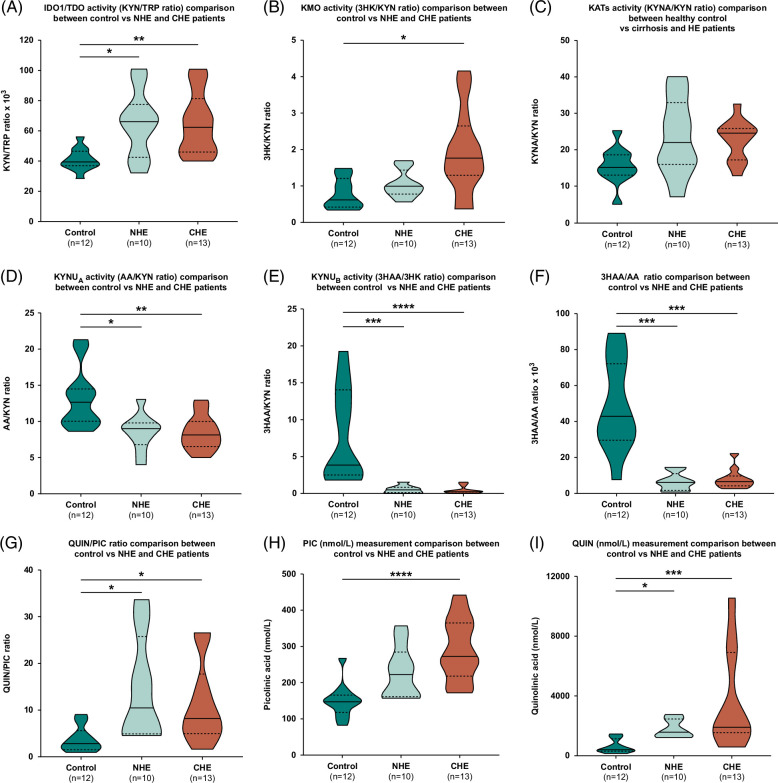
Comparison of KP metabolites and enzyme activity in controls, patients with NHE and CHE. (A) IDO-1/TDO (KYN/TRP ratio), (B) KMO (3HK/KYN ratio) (C) KATs (KYNA/KYN ratio), (D) KYNU A (AA/KYN ratio), (E) KYNU B (3HAA/3HK ratio), (F) 3HAA/AA ratio, (G) QUIN/PIC ratio, (H) PIC (nmol/L), (I) QUIN (nmol/L). Statistical significance was denoted as *p<0.05; **p<0.01; ***p<0.001; ****p<0.0001 using a one-way ANOVA test with the Kruskal-Wallis multiple comparison test. Abbreviations: 3HAA, 3-hydroxy anthranilic acid; 3HK, 3-hydroxykynurenine; AA, anthranilic acid; CHE, covert hepatic encephalopathy; IDO-1, indoleamine 2,3-dioxygenase; KAT, Kynurenine aminotransferase; KYN, kynurenine; KYNA, kynurenic acid; PIC, picolinic acid; QUIN, quinolinic acid; NHE, no hepatic encephalopathy; TDO, tryptophan 2,3-dioxygenase; TRP, tryptophan.

KP activation is defined by higher KYN/TRP ratios, which infers an upregulation of IDO-1 and tryptophan 2,3-dioxygenase (TDO), the rate-limiting enzymes of the pathway. IDO-1 and TDO enzyme activity was significantly higher in patients with CHE (65.04±20.72, *p*=0.003) and NHE (64.85±22.10, *p*=0.015) compared to HC (40.95±7.301), but there was no significant difference between CHE and NHE groups (Figure 3A). This was primarily driven by lower TRP concentration in both CHE (24.93 μM±9.48, *p*=0.001) and NHE (21.78 μM±4.28, *p*<0.001) patients in comparison to HC (39.73 μM±5.89). There was no significant difference in KYN concentration among the 3 cohorts. Table 2 and Supplemental Table S1, http://links.lww.com/HC9/B63, detail all KP enzyme activity ratios and metabolite concentrations for the 3 groups.

**TABLE 2:** Activity of KP enzymes measured in the plasma of healthy control, patients with NHE and CHE

	Healthy control and patient groups			Kruskal-Wallis test—adjusted *p*		
Enzymes	Healthy control (HC) (mean±SD; n=12)	No hepatic encephalopathy (NHE) (Mean±SD; n=10)	Covert hepatic encephalopathy (CHE) (Mean±SD; n=13)	NHE vs. HC	CHE vs. HC	CHE vs. NHE
KYN/TRP ratio × 100 (IDO-1/TDO)	40.95±7.301	64.85±22.10	65.04±20.72	***0.015***	***0.003***	>0.99
3HK/KYN ratio (KMO)	0.7889±0.4250	1.050±0.3906	1.944±1.158	0.93	***0.017***	0.34
AA/KYN ratio (KYNU_A_)	13.19±4.221	8.521±2.529	8.397±2.382	***0.016***	***0.006***	>0.99
KYNA/KYN ratio (KAT)	15.68±5.086	23.48±10.62	22.74±5.497	0.087	0.053	>0.99
3HAA/3HK ratio (KYN U_B_)	7.862±6.389	0.5690±0.4754	0.5158±0.5067	***<0.001***	***<0.001***	>0.99
3HAA/AA ratio	48.62±25.26	6.051±5.049	8.008±5.444	***<0.001***	***<0.001***	>0.99
QUIN/PIC ratio	3.713±2.731	14.33±11.53	11.33±8.772	***0.019***	***0.040***	>0.99

Bold Values are statistically significance *p*-values.

Abbreviations: 3HAA, 3-hydroxyanthranilic acid; 3HK, 3-hydroxykynurenine; AA, anthranilic acid; CHE, covert hepatic encephalopathy; IDO1, indoleamine 2,3-dioxygenase; KAT, kynurenine aminotransferase; KMO, kynurenine 3-monooxygenase; KYN, kynurenine; KYNA, kynurenic acid; KYNU, kynureninase; NHE, no hepatic encephalopathy; PIC, picolinic acid; QUIN, quinolinic acid; TDO, tryptophan 2,3-dioxygenase; TRP, tryptophan.

### Kynurenine monooxygenase enzyme activity is elevated in patients with CHE but not in patients with NHE

KYN can be catabolized to 3HK, AA, or KYNA by the enzymes kynurenine monooxygenase (KMO), kynureninase (KYNU) or kynurenine aminotransferase (KATs), respectively, as shown in Figure 2. Patients with CHE demonstrated a significantly higher level of KMO activity, as inferred by 3HK/KYN ratio, in comparison to HC (1.94±1.16 vs. 0.79±0.43; *p*=0.017). These findings are graphically represented in Figure 3B and were primarily due to higher concentrations of 3HK in patients with CHE (2.97 nM±2.01) versus patients with NHE (1.39 nM±0.57) and HC (1.88 nM±1.35); *p*>0.05 for all comparisons.

There was no significant difference between groups in KAT activity among the groups, as represented by the KYNA/KYN ratio (Figure 3C). However, KAT activity tended to be higher in patients with CHE (22.74±5.50, *p*=0.053) and NHE (23.48±10.62, *p*=0.087) in comparison to HC (15.68±5.09); *p*>0.05 for all comparisons.

Patients with both CHE and NHE demonstrated reduced KYNU A activity in comparison to HC, as inferred by significantly lower AA/KYN ratios (CHE 8.40 ± 2.38 vs. NHE 8.52 ± 2.53 vs. HC 13.19 ± 4.22, *p*=0.006 for CHE vs. HC and *p*=0.016 for NHE vs. HC). These findings are graphically represented in Figure 3D and were primarily due to lower concentrations of AA in patients with CHE (13.47 nM±6.30, *p*=0.041) and NHE (11.40 nM±3.99, *p*=0.010) in comparison to HC (23.91 nM±12.95).

### Dysregulation of the main route of the KP in patients with CHE and NHE

Regarding the downstream metabolites, kynureninase B (KYNU B) activity is represented by the ratio of 3HAA/3HK, and the 2 metabolites at the final node of the pathway QUIN and PIC. KYNU B enzyme activity, measured as the 3HAA/3HK ratio, was significantly lower in patients with CHE (0.52±0.51, *p*<0.001) and NHE (0.57±0.48, *p*=0.001) as compared to the HCs (7.86±6.39). There was no significant difference between the CHE and NHE groups. These findings are graphically represented in Figure 3E and were primarily due to lower concentrations of 3HAA in patients with CHE (1.07 nM±0.66, *p*=0.001) and NHE (0.75 nM±0.77, *p*<0.001) in comparison to HC (11.13 nM±7.21). As represented in Figure 3F, 3HAA/AA ratios were also significantly lower in patients with CHE (8.01± 5.44, *p*=0.001) and NHE (6.05±5.05, *p*<0.001) versus HCs (48.62±25.26). There was again no significant difference between the CHE and NHE groups.

There was a trend toward higher PIC concentrations in patients with CHE (290.80 nM±86.40) and NHE (228 nM±71.56) versus HC (149.4 nM±46.65), with a statistically significant difference between patients with CHE and HC only (*p*<0.001). There was a similar trend when observing QUIN concentrations in the 3 groups (CHE 3726 nM±3385 vs. NHE 1788 nM±632.3 vs. HC 624 nM±457; *p*<0.001 for CHE vs. HC and *p*=0.032 for NHE vs. HC). Finally, we observed that the QUIN/PIC ratio was significantly higher in patients with both CHE (11.33±8.77, *p*=0.040) and NHE (14.33±11.53, *p*=0.019) in comparison to HC (3.71±2.73). These results are graphically represented in Figure 3G–I.

### Correlations between KP enzymes/metabolites and clinical parameters in patients with CHE

**FIGURE 4:**
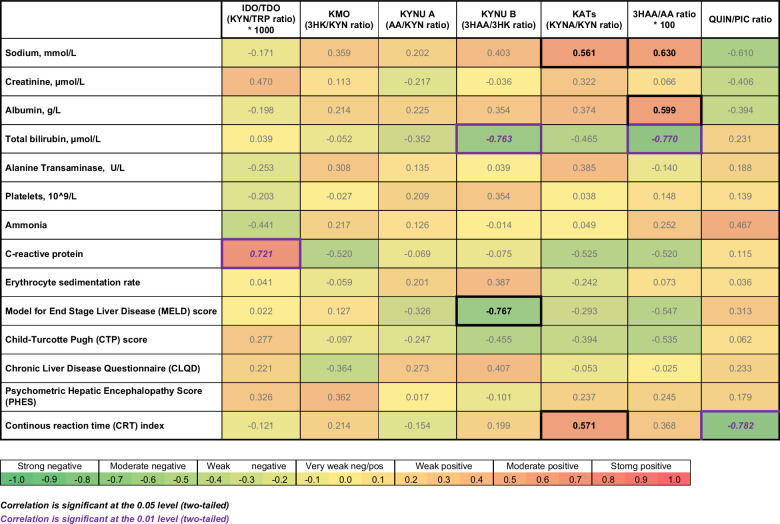
Heatmap of Spearman correlation analysis between KP enzymes and clinical parameters in patients with CHE. Shades of red denote positive correlations, while shades of green represent negative correlations. Correlations with a *p-*value ≤0.05 are marked in black font, and those with a *p-*value ≤0.01 are highlighted in purple italic font. Abbreviations: 3HAA, 3-hydroxy anthranilic acid; 3HK, 3-hydroxykynurenine; AA, anthranilic acid; CLQD, chronic liver disease questionnaire; CRT, continuous reaction time; CTP, Child-Turcotte-Pugh; IDO-1, indoleamine 2,3-dioxygenase; KAT, Kynurenine aminotransferase; KYN, kynurenine; KYNA, kynurenic acid; PIC, picolinic acid; QUIN, quinolinic acid; TDO, tryptophan 2,3-dioxygenase; TRP, tryptophan.

Spearman correlation analysis demonstrated multiple statistically significant correlations between the KP enzymes and clinical markers in patients with CHE, as presented in Figure 4. IDO-1/TDO activity demonstrated a moderately positive correlation with inflammatory marker c-reactive protein (R_s_=0.721, *p* ≤ 0.01). Median C-reactive protein in patients with CHE was 6.8, with a range from 0.3 to 34.7. KYNU B activity, represented by the 3HAA/3HK ratio, demonstrated moderately negative correlations with bilirubin (R_s_=−0.763, *p* ≤ 0.01) and MELD score (R_s_=-0.767, *p* ≤ 0.05). KATs activity, represented by KYNA/KYN ratio, demonstrated moderately positive correlations with sodium (R_s_=0.561, *p* ≤ 0.05) and CRT index (R_s_=0.571, *p* ≤ 0.05). 3HAA/AA ratio demonstrated a moderately negative correlation with bilirubin (R_s_=−0.770, *p* ≤ 0.01) and a moderately positive correlation with sodium (R_s_=-0.630, *p* ≤ 0.05) and albumin (R_s_=0.599, *p* ≤ 0.05). Finally, QUIN/PIC ratio demonstrated a moderately negative correlation with CRT index (R_s_=−0.782, *p* ≤ 0.01). While this study focuses on correlations with KP enzymes and metabolite ratios, correlations pertaining to specific KP metabolites are also summarized in a heatmap in Supplemental Figure S1, http://links.lww.com/HC9/B64.

## DISCUSSION

A growing body of evidence suggests that neuroinflammation mediated by chronic hyperammonemia and proinflammatory cytokines plays a crucial role in CHE.^11^,^38^ Neuroinflammation plays a key role in the dysregulation of the KP, which has also been implicated in neurodegenerative conditions (eg, Alzheimer disease), neurological autoimmune diseases (eg, multiple sclerosis), and psychiatric disease (eg, major depression).^39^ This study is the first to highlight that the KP is dysregulated and skewed toward the production of neurotoxic metabolites in patients with CHE and that the activity of the pathway enzymes is closely associated with the clinical parameters of CHE.

**FIGURE 5:**
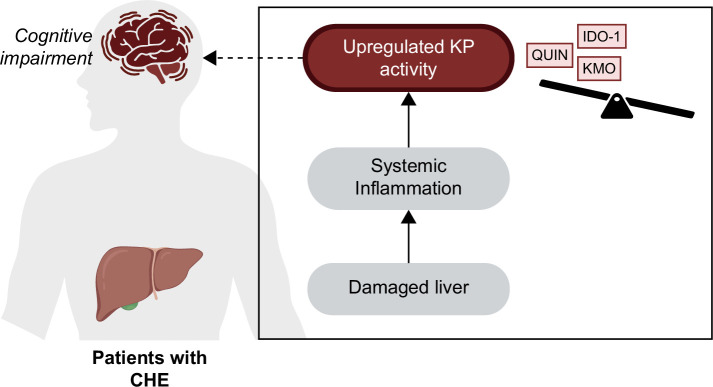
Summarizing the links between KP dysregulation and cognitive impairment in patients with CHE. Abbreviations: CHE, covert hepatic encephalopathy; IDO-1, indoleamine 2,3-dioxygenase; KMO, kynurenine 3-monooxygenase; QUIN, quinolinic acid.

Our study demonstrates that the KP is highly activated in patients with cirrhosis, both with and without CHE Figure 5. This is inferred through a decrease in TRP levels and corresponding elevation in IDO-1/TDO enzyme activity. These findings are all in keeping with the characterization of cirrhosis as an inflammatory process^7^ driven by cytokine release from KCs and hepatocytes.^40^ As aforementioned, KP-dependent induction of IDO1 has been demonstrated in HE animal models^27^,^28^ and patients with OHE^30^,^31^ either via elevation of TRP or downstream metabolites. Moreover, Jiang et al^28^ demonstrated that the administration of IDO-1 inhibitors resulted in reversal of behavioral changes in BDL rats. As such, IDO-1 inhibitors serve as a promising therapeutic target, having also demonstrated value in ameliorating liver fibrosis and targeting cancer immune escape.^41^,^42^ Compared to IDO-1, the role of TDO enzyme in liver disease remains understudied despite being the prominent KP enzyme under normal physiological conditions. Thus, the role of TDO enzyme in cirrhosis and CHE needs further clarification.

We found that KMO enzyme activity, represented by the 3HK/KYN ratio, is significantly upregulated in patients with CHE. This likely owes to systemic IDO-1 activation shifting the KP toward the production of 3HK, leading to elevated levels of the neurotoxic QUIN over the neuroprotective KYNA.^39^ Our study also demonstrates a statistically significant stepwise trend in QUIN concentrations when comparing HCs to patients with NHE and CHE, as was also found in prior HE animal models.^27^ QUIN also has the capacity to generate reactive oxygen species and inflict neuronal excitotoxicity via overactivation of N-methyl-D-aspartate receptors.^15^,^16^,^17^ In patients with CHE and NHE, higher QUIN levels correspond with lower 3HAA levels, likely reflecting the brisk catabolism of 3HAA to downstream metabolites QUIN and PIC. Furthermore, we found that the 3HAA/AA ratio was significantly reduced in patients with CHE and NHE versus HCs, as is the case in other disease states of inflammation and neurological disorders.^43^ Darlington and colleagues propose that reduced 3HAA/AA ratio may be a compensatory mechanism in keeping with observed effects of antagonization of QUIN toxicity, protection against oxidative stress, and reduction in inflammatory response.

Our study also demonstrates higher levels of neuroprotective metabolites KYNA and PIC in patients with CHE and NHE compared to HCs, with only the comparison of PIC between patients with CHE and controls reaching statistical significance. KYNA is an N-methyl-D-aspartate antagonist that blocks the excitotoxic effects of QUIN, but there are insufficient levels to block QUIN binding during the disease state.^20^,^39^ Although a stepwise increase in KYNA levels is apparent when comparing HCs to NHE and then CHE, we were not able to demonstrate statistically significant results, and KATs enzyme activity was similar between groups. PIC has neuroprotective and antiproliferative properties, and our finding of increased concentrations in patients with CHE may represent a compensatory effect.^44^ Production of PIC is mediated by the 2-amino 3-carboxymuconate 6-semialdehyde decarboxylase enzyme, which is present in the liver and kidney and may be upregulated in the setting of chronic hepatic inflammation. Previous studies have also reported an increased production of PIC in chronic liver diseases such as chronic hepatitis C infection.^45^ Interestingly, we found that QUIN/PIC ratio was significantly higher in patients with CHE and NHE compared to HCs. While this ratio is not a marker of any particular enzyme activity, it suggests that the higher PIC concentrations in patients with CHE and NHE are insufficient to compensate for the respective rise in QUIN concentrations, implying an overall consequence of neurotoxicity in these individuals.

In addition to highlighting the contributory role of the KP in CHE, our study demonstrates clinically relevant correlations between KP enzymes and biochemical markers of inflammation and end-stage liver disease. In patients with CHE, higher IDO-1/TDO activity was correlated with higher C-reactive protein, which is well known as a surrogate marker for inflammation and affirms its central role in the HE hypothesis. Previous studies have demonstrated a correlation between neuropsychometric test scores and biochemical markers of inflammation in patients with cirrhosis.^46^ Importantly, the correlation between KP activation and inflammation was not reproduced in the analysis of patients with NHE. Furthermore, lower 3HAA/AA ratios and KATs activity, the latter indicative of limited production of neuroprotective KYNA, were associated with hyponatremia in patients with CHE. Hyponatremia is a known consequence of cirrhosis and portal hypertension and has been found to be a strong predictor of electroencephalogram changes and OHE in patients with cirrhosis.^47^,^48^ In the same patient group, hyponatremia was also associated with higher QUIN levels and PIC levels, the latter of which may be in keeping with a compensatory mechanism.^44^ Moreover, various markers included in the CP score were also found to correlate with the activity of certain KP enzymes in patients with CHE only. Lower albumin levels were linked with lower 3HAA/AA ratios, and higher bilirubin levels were linked with lower 3HAA/AA ratios and low KYNU B activity. While the CP score did not correlate with the activity of any KP enzymes, higher MELD scores were associated with low KYNU B activity in patients with CHE, which possibly reflects a brisk shift toward the production of downstream neurotoxic metabolites, as earlier discussed.

The links between the KP pathway and other considerations, such as psychometric scores and quality of life, were less clear. In patients with CHE, better CRT index scores were associated with a shift of balance toward the production of neuroprotective metabolites, inferred by higher KAT activity and lower QUIN/PIC ratios. However, while the PHES score was negatively correlated with KMO and KAT activity in patients with NHE, it was not associated with any KP metabolites or enzyme activity in patients with CHE. These findings are difficult to interpret and perhaps suggest that any clinical impacts of KP dysregulation are distinct from the cognitive domains encompassed by the PHES score.^34^ Chronic Liver Disease Questionnaire scores were not correlated with KP enzyme activity in patients with either CHE or NHE, suggesting that KP dysregulation may not impact patient’s disease perception. This is despite Chronic Liver Disease Questionnaire scores demonstrating a correlation with the severity of liver disease in a previous cohort study.^49^

The predominant limitation of our study is its small cohort size, which likely contributed to most comparisons between patients with CHE and NHE not reaching statistical significance. An alternative explanation for this is that KP dysregulation may precede the clinical manifestations of CHE. This would be in keeping with our previous findings in the same cohort that even patients with NHE demonstrate a degree of cerebral metabolite derangement and increased blood-brain barrier permeability.^10^ Similarly, the correlations between KP metabolites and biochemical/clinical markers should also be revisited in a larger study. Unfortunately, no Aboriginal and Torres Strait Islander people were included in the study, again owing to the small sample size, which limits the generalizability of the results for an Australian population. Moreover, there was no clinical data available for the age and gender-matched HCs, which were sourced from an external cohort. We acknowledge that age may influence the differences observed between the patients and HCs, which would be an important consideration for future studies. Also of note, we measured serum levels of KP metabolites rather than CSF levels, given the ethics limitations of our study. However, CSF and serum KP metabolite levels have previously been shown to correlate strongly in a HE animal study,^27^ probably due to increased blood-brain barrier permeability in this disease state. Moreover, the majority of patients with CHE had already initiated treatment with Lactulose, which could also influence KP regulation. Lactulose has been shown to reduce levels of indoxyl sulfate, a gut-derived toxic metabolite of TRP via the indole pathway, in select animal studies,^50^ but its impact on KP metabolites is unclear. Finally, while many of these findings are novel, and we hypothesize that the KP pathway may contribute to the neuropsychiatric abnormalities of CHE, these correlations cannot be used to support direct causal relationships.

In conclusion, we demonstrate that KP is highly activated in patients with CHE and that dysregulation of the pathway becomes evident prior to the clinical onset of CHE in patients with cirrhosis. KP activation is associated with biochemical measures of inflammation and other poor prognostic markers of cirrhosis but does not correlate well with neuropsychometric testing or patient perception of disease. The severity of liver disease may dictate the extent of compensatory increase in neuroprotective metabolites in the pathway. These findings should be validated in larger studies but provide preliminary support for therapeutics targeting KP dysregulation.

## Supplementary Material

SUPPLEMENTARY MATERIAL
