# Supplementary material for: Activation of the kynurenine pathway identified in individuals with covert hepatic encephalopathy
Source: Hepatol Commun. 2024 Nov 15;8(12):e0559. doi: 10.1097/HC9.0000000000000559 (PMC11567712; doi:10.1097/HC9.0000000000000559)
Supplement: SUPPLEMENTARY MATERIAL [file hc9-8-e0559-s001.docx]

|  | *Healthy control and patient groups* | | | *Kruskal-Wallis test (2-way ANOVA results) - adjusted P-value* | | |
| --- | --- | --- | --- | --- | --- | --- |
| Metabolites | Healthy control (HC)  (Mean ± SD; n=12) | No hepatic encephalopathy (NHE)  (Mean ± SD; n=10) | Covert hepatic encephalopathy (CHE)  (Mean ± SD; n=13) | NHE vs HC | CHE vs HC | CHE vs NHE |
| TRP µM | 39.73 ± 5.891 | 21.78 ± 4.278 | 24.93 ± 9.479 | ***<0.001*** | ***0.001*** | 0.75 |
| KYN µM | 1.638 ± 0.4360 | 1.343 ± 0.2819 | 1.575 ± 0.7716 | 0.49 | >0.99 | >0.99 |
| KYNA nM | 22.86 ± 8.225 | 31.03 ± 16.19 | 35.99 ± 19.34 | 0.94 | 0.14 | >0.99 |
| 3HK nM | 1.882 ± 1.354 | 1.385 ± 0.5740 | 2.973 ± 2.014 | 0.89 | 0.83 | 0.12 |
| AA nM | 23.91 ± 12.95 | 11.40 ± 3.991 | 13.47 ± 6.303 | ***0.010*** | ***0.041*** | >0.99 |
| 3HAA nM | 11.13 ± 7.213 | 0.7500 ± 0.7669 | 1.070 ± 0.6556 | ***<0.001*** | ***<0.001*** | >0.99 |
| PIC nM | 149.4 ± 46.65 | 228 ± 71.56 | 290.8 ± 86.40 | 0.053 | ***<0.001*** | 0.32 |
| QUIN nM | 624 ± 457 | 1788 ± 632.3 | 3726 ± 3385 | ***0.032*** | ***<0.001*** | >0.99 |

**Supplementary Table 1:** Levels of the KP metabolites measured in the plasma of healthy control, NHE and CHE patients.

**Abbreviations:** TRP: Tryptophan; KYN: Kynurenine; KYNA: Kynurenic acid; 3HK: 3-hydroxykynurenine; AA: Anthranilic acid; 3HAA: 3-hydroxyanthranilic acid;

PIC: Picolinic acid; QUIN: Quinolinic acid
