# Supplementary material for: Activation of the kynurenine pathway identified in individuals with covert hepatic encephalopathy
Source: Hepatol Commun. 2024 Nov 15;8(12):e0559. doi: 10.1097/HC9.0000000000000559 (PMC11567712; doi:10.1097/HC9.0000000000000559)
Supplement: SUPPLEMENTARY MATERIAL [file hc9-8-e0559-s002.docx]

******

***Correlation is significant at the 0.05 level (two-tailed)***

***Correlation is significant at the 0.01 level (two-tailed)***

**Supplementary Figure 1: Heatmap of Spearman’s correlation analysis between KP metabolites and clinical parameters in CHE patients.** Shades of red denote positive correlations, while shades of green represent negative correlations. Correlations with a *p-*value ≤ 0.05, are marked in black font, and those with a *p-*value ≤ 0.01 are highlighted in purple italic font.
